# Supplementary material for: Investigating stronger tolerant network against cascading failures in focusing on changing degree distributions
Source: PLoS One. 2024 Jul 10;19(7):e0297094. doi: 10.1371/journal.pone.0297094 (PMC11236162; doi:10.1371/journal.pone.0297094)
Supplement: Attachment — Submitted filename: Response to Reviewers.pdf [file pone.0297094.s008.pdf]

I hope this message finds you well. Thank you for taking time to review our manuscript titled "Investigating stronger tolerant network against cascading failures in focusing on changing degree distributions" submitted to PLOS ONE under Manuscript ID PONE-D-23-15565. We greatly appreciate your insightful feedbacks, which have significantly contributed to improving the quality of our work.

We have carefully considered each of your comments and suggestions, and we are pleased to submit the revised version of our manuscript along with this response letter outlining the changes we have made in response to your feedback.

We explain the revisions made:

For Reviewer #1

Q1. The main concern about this study is that the changing degree distributions of network influence the cascading failures are not modeled in the cascading failures model?

A1. Since the word "model" in our previous version might have been misleading, it is replaced to "process" rather than modeling cascading failures. We apologize for any confusion.

Q2. What does the result "In addition, the networks have high efficiency similar to SF networks" in the Abstract specifically mean?

A2. We have removed the insufficient part from the abstract.

Q3. There are several abbreviations that require explanation, for example, BA.

A3. We have added an explanation about Barabási Albert (BA) in section "Continuously changing degree distribution."

For Reviewer #2

Q1. The abstract is too long and contains too many details that should be moved to the introduction or the methods section. Please shorten the abstract to about 150 words and focus on the main motivation, results and implications of your work.

A1. The abstract is shorten in 64 words.

Q2. The introduction lacks a clear statement of the research problem and the research gap that your paper aims to fill. Please provide more background information on the

existing literature on network robustness and cascading failures, and explain how your work differs from or extends previous studies.

A2. In order to bridge bring a fresh perspective on the interplay of degree distributions, loops, and tolerance against cascading failures, the guideline is given in the section "Introduction".

Q3. The methods section is not well explained and lacks some important information. For example, how did you generate the networks with different degree distributions? What are the parameters and assumptions of your cascading failure model? How did you measure the efficiency of the networks? Please provide more details and references for your methods and justify your choices.

A3. We added the detailed explanations of how to generate the networks in line 69-73 on the section "Continuously changing degree distributions". In addition, the tolerance parameter α included in Motter model on simulation is described in section "Result", line 174, and for network efficiency calculated by Eq. (4) in the section "Cascading failures".

Q4. The results section is too descriptive and does not provide enough analysis and interpretation of your findings. For example, why do networks with narrower degree distributions have higher tolerance and efficiency than scale-free networks? What are the underlying mechanisms or principles that explain this phenomenon? How do your results compare with other studies on network robustness and cascading failures? Please discuss these aspects more thoroughly and provide some figures or tables to illustrate your results.

A4. An explanation of why networks with narrower degree distributions are more tolerant than scale-free networks is found in lines 186 - 214. Additionally, the efficiency comparison is described in lines 215 -223. To explain the mechanism and principle, some sentences have been added in lines 186 - 223. Furthermore, network efficiency is closely related to the average shortest path length, which is discussed in lines 105-113 on section "Continuously changing degree distributions". Finally, existing studies about cascading failures concentrates on the pin-points of SF networks and ER random graphs. A comparison between them and the networks with narrower degree distributions of this study can be found in lines 186 - 214 as noted earlier.

Q5. Cascading failures is a complex process, and under the same attack, the evolution of cascading failures can have multiple possible paths. How does your research take this into account? And please elaborate on the multiple evolution processes of network cascading failures under three different attacks.

A5. In this study, we are choosing routes deterministically. Considering that there's also the option of randomly choosing from multiple shortest paths, I have added the sentence "Remember that, after removing either AN=1 or 50 nodes as triggers, the flows between and are equally distributed to multiple shortest paths of a same length by Eq. (1) in the determinant choosing. Therefore, stochastic choosing from multiple shortest paths is beyond our scope of this study.", lines 157-159 on the section "Cascade failures".

Q6. In my opinion, for the resilience of complex networks, it is not that the narrower the degree distribution, the better, nor is it the wider the better. Instead, it is about finding its balance point. Your article does not explain this part very clearly. How narrow should the degree distribution be for optimal resilience?

A6. It is suggested that a random regular graph with 0 width has the optimal robustness of connectivity. To clarify the optimal tolerance for cascading failures, perturbation analysis will be required with huge numerical computations as a part of future works. It is added in lines 244-246 on the section "Conclusion".

For Editor

1. Style

We have ensured the revision met the style requirements. The template is in the following URL. https://journals.plos.org/plosone/s/latex

2. Acknowledgments

According to your suggestion, the Acknowledgments Section has been replaced manuscript. The Funding statement remains unchanged from the current version.

3. Upload Figures

Fig 1, 4, and 5 are uploaded.

We believe that the revisions made have strengthened the overall content and improved the presentation of manuscript. We are confident that the updated version meets the high standards of PLOS ONE and addresses the concerns raised during the review process.

Once again, we would like to express our gratitude for your valuable feedback and guidance. We believe that your suggestions have significantly contributed to enhancing the quality of our work. Please find attached the revised manuscript marked with changes for your convenience.

Thank you for spending your time and consideration.

Sincerely,

Ryota Kusunok
Graduate student
Japan Advanced Institute of Science and Technology
ryotakusunoki@hotmail.com
+8190-7791-9989
