# Supplementary material for: Investigating stronger tolerant network against cascading failures in focusing on changing degree distributions
Source: PLoS One. 2024 Jul 10;19(7):e0297094. doi: 10.1371/journal.pone.0297094 (PMC11236162; doi:10.1371/journal.pone.0297094)
Supplement: Attachment — Submitted filename: Response_to_Reviewer.pdf [file pone.0297094.s009.pdf]

Thank you for taking time to review our manuscript titled "Investigating stronger tolerant network against cascading failures in focusing on changing degree distributions" submitted to PLOS ONE under Manuscript ID PONE-D-23-15565. We greatly appreciate your insightful feedbacks, which have significantly contributed to improving the quality of our work.

We have carefully considered each of your comments and suggestions, and we are pleased to submit the revised version of our manuscript along with this response letter.

We explain the revisions made:

For Reviewer #1

Q1 means that how does link betweenness centralities affect the spread of cascading failures by affecting node load Li(t) ? This is suggested to be further explained in the section on Cascading failures. In addition, is the value of Li, gst and gst(i) changed with the spread of cascading failures? Li(0) should also be explained.

For the 1st and 2nd questions, my answers are NO and YES, respectively. Thus, I have added a sentence on lines 137-139 explaining that link betweenness centrality is used to measure the amount of bypass routes. L_i, g_{st}, $g_{st}(i)$ are recalculated at each step of the cascading failure simulation, which I have noted on lines 139. Additionally, I have included a description of $L(0)$, the initial load of each node, on lines 141-142.

For Reviewer #2

Q1. In the abstract, it may be better to avoid using abbreviations such as SF and LCC, as they may not be familiar to all readers. Instead, spell out the full terms or use more general descriptions.

I have avoided using abbreviations in the abstract according to the suggestion.

Q2. In the introduction, it may be helpful to briefly explain what cascading failures are and why they are important to study, as some readers may not be familiar with this concept.

I have added the explanation about cascading failures and their significance on lines 50-57.

Q3. In the methods section, it may be useful to provide some examples or references for real-world networks that have power-law, exponential, or narrower degree distributions, to motivate the choice of models and parameters.

I have added real-world examples of power-law distributions on lines 3-7. For exponential and narrower distributions, please refer to lines 38-41. Each of these additions can be found in the Introduction.

Q4. In the results section, it may be interesting to compare the network efficiency before and after cascading failures, to see how much efficiency is lost due to the damage. It may also be worth discussing how the network efficiency relates to other network properties, such as clustering coefficient or average path length.

According to the suggestion, I have added Fig 6 that shows the network efficiency E before and after cascading failures triggered by localized random, max-degree, and max-load node removals in randomized networks after generations by PA and IPA in changing degree distribution. In addition, we mention that the relationship between efficiency and other network properties, such as the clustering coefficient, remains a topic for future works.
