# Supplementary material for: Investigating stronger tolerant network against cascading failures in focusing on changing degree distributions
Source: PLoS One. 2024 Jul 10;19(7):e0297094. doi: 10.1371/journal.pone.0297094 (PMC11236162; doi:10.1371/journal.pone.0297094)
Supplement: Attachment — Submitted filename: Review letter for Editor.docx [file pone.0297094.s007.docx]

Dear Editor,

Thank you for sending me the manuscript "Investigating stronger tolerant network against cascading failures in focusing on changing degree distributions" by Ryota Kusunoki et al. for review. The manuscript presents a numerical study of the effects of degree distribution on the robustness and efficiency of networks under cascading failures. The authors claim that networks with narrower degree distributions are more tolerant to cascading failures and have high efficiency similar to scale-free networks. The manuscript is well-written and the topic is interesting and relevant. However, I have some major and minor concerns that need to be addressed before the manuscript can be accepted for publication.

Major issues:

1. The authors do not provide a clear definition of what they mean by "narrower degree distributions". How do they measure the width or variance of the degree distribution? What are the criteria for choosing the parameters of the degree distributions? How do they compare different types of degree distributions, such as Poisson, exponential, or power-law?

2. The authors do not explain how they model cascading failures in their networks. What are the rules for removing nodes and links after an initial attack? How do they measure the damage or size of the cascade? How do they ensure that their results are robust and not dependent on the choice of initial attack or random seed?Cascading failures is a complex process, and under the same attack, the evolution of cascading failures can have multiple possible paths. How does your research take this into account? And please elaborate on the multiple evolution processes of network cascading failures under three different attacks.

3. The authors do not provide sufficient evidence or analysis to support their claim that networks with narrower degree distributions have high efficiency similar to scale-free networks. They only show one figure (Fig. 4) that compares the average shortest path length of different networks, but this is not enough to capture the complexity and diversity of network efficiency. They should also consider other measures, such as betweenness centrality, clustering coefficient, modularity, or spectral properties. In my opinion, for the resilience of complex networks, it is not that the narrower the degree distribution, the better, nor is it the wider the better. Instead, it is about finding its balance point. Your article does not explain this part very clearly. How narrow should the degree distribution be for optimal resilience?

Minor issues:

4. The abstract is too long and contains too much technical detail. It should be concise and highlight the main findings and contributions of the paper. The authors should also avoid using abbreviations (e.g., SF) that are not defined or explained in the abstract.

5. The introduction is too broad and does not provide a clear motivation or research gap for the study. The authors should focus more on the specific problem of cascading failures in networks and how their work addresses it. They should also review the relevant literature more critically and discuss how their approach differs from or improves upon previous studies.

6. The figures are not well-designed and labeled. They should have clear titles, captions, legends, axes, and units. They should also use consistent colors and symbols for different types of networks or parameters. Some figures (e.g., Fig. 2) are too small and hard to read.

7. The references are incomplete and inconsistent. Some references are missing important information, such as year, volume, issue, or page numbers. Some references use different styles or formats, such as APA, IEEE, or Harvard. The authors should follow the journal's guidelines for referencing and citation.

I hope that my comments will help the authors improve their manuscript. I look forward to seeing a revised version of the paper.

Sincerely,

Dr. Zack Yang
